# A Glance at Molecular Advances in Cancer Genetics: A Baffling Puzzle Still to Be Solved

**DOI:** 10.3390/ijms24021394

**Published:** 2023-01-11

**Authors:** Paola Ghiorzo, William Bruno

**Affiliations:** 1 Genetics of Rare Cancers, IRCCS Ospedale Policlinico San Martino, Largo Rosanna Benzi X, 16132 Genoa, Italy; 2 Department of Internal Medicine and Medical Specialties (DiMI), University of Genoa, Viale Benedetto XV 6, 16132 Genoa, Italy

The purpose of this first Special Issue is to provide a glance at the molecular advances in cancer genetics to untangle the complexity of tumorigenesis.

In the last two decades, massive parallel sequencing techniques sped up the identification of germline and somatic variants, ripping and segmenting the molecular profiles of a wide range of cancers and, as a result, their classification and nomenclature. 

Therefore, the combination of germline testing and tumor mutational assessments has helped us to discern the clinical and also therapeutic relevance of the identified variants. Hereditary predisposition and translational cancer genomics were previously considered to be separate, but now their combination provide us with the opportunity to improve the identification of actionable germline and somatic variants and our understanding of the genetic and molecular bases of cancer.

A concerning challenge in the field of cancer susceptibility is that of missing heritability. However, the identification of pathogenic variants with clinically suggestive histories needs to be supported by evidence in order to confirm their actual role.

Finding a pathogenic variant through a multigene panel should not convey the false message that a conclusive answer was found.

The study from Zuntini et al. [1] tackles the issue of the premature inclusion of candidate genes in multigene panel testing. For example, *NBN* is involved in homologous recombination deficiency (HRD), and it is allegedly associated with a higher risk of breast cancer.

Their results suggest that the preliminary findings are insufficient to justify testing specific genes in a diagnostic setting in the absence of case–control, functional, and segregation analyses. Hence, we need diagnostic multigene panels that have been constructed using solid evidences.

HRD, a well-known feature of many cancers, also became of interest for its therapeutic implications [2], e.g., PARP-inhibitors (PARPi) sensitivity.

As described in the paper from Sahnane et al. [3], aside from the known causes of HRD in epithelial ovarian cancer (EOC), which are represented by germline or somatic variants in *BRCA1/2*, the mechanisms with a well-known role in gene expression are often not sufficiently investigated or included in clinical practice e.g., epigenetic mechanisms such as promoter methylation. Therefore, the aforementioned study investigates the involvement of this mechanism in HRD in a series of EOCs without *BRCA1/2* germline mutations. Notably, it identifies a subgroup of patients who can potentially benefits from PARPi. These results will prompt other clinical studies which clarify the predictive role of somatic *BRCA* methylation of the PARP therapy response.

Wadowska et al.’s review [4] also broadens this perspective, emphasizing that the research of clinically useful biomarkers should include methylation analyses and microsatellite analysis or miRNA expression profiles to achieve the early diagnosis of common cancers. In particular, for lung cancer, although miRNA profiles seem to have the highest diagnostic potential, the standardization for their implementation in clinical practice is still missing.

Urazbakhtin et al. and Choi et al., respectively, include more cryptic and neglected aspects of tumorigenesis, e.g., retrotranspositional activity in child and adult acute lymphloblastoid leukemia or oncogenic hotspot variants identification, such as that in *GNAQ* in the hepatocellular carcinoma [5,6].

For clinical and therapeutic purposes, next-generation sequencing (NGS) technologies are still used for the routine analysis of a few well-established genes. For example, Dameri et al. [7] researched the predictive utility of NGS performed on different types of tissues to identify the actionable molecular targets e.g., the HRD of tumoral mutational burden in metastatic triple-negative breast cancer (mTNBC).

However, tumoral heterogeneity is a problem that has been faced by three studies using a multiomics perspective.

The paper by Bresadola et al. [8] using a case of synchronous cancers shows how the integration between genomic and transcriptomic analyses may contribute to a personalized revision of the therapeutic management, dissecting, and profiling of a tumor by germline variants, the immunological microenvironment, and cellular clones.

The review of single cell genomic studies by Rontauroli et al. [9] broadens this view by dissecting clonal architecture and the mutation acquisition order accuracy at different omics levels. The study by Carretta et al. [10] actually proposes a novel single-cell genomics approach for hematological malignancies to demonstrate how it overcomes the limitations of bulk sequencing in terms of the correct proliferation of clonal phylogeny.

As we are beginning to unravel the complexity of each cancer type, the classical genetic multistep model of tumorigenesis appears to be increasingly insufficient.

Pastorino et al.’s review [11] initially uses similar assumptions that are applied to rare tumors.

It is extremely difficult to create a comprehensive model of tumorigenesis because of diversity of the site of origin, the molecular profiles, and the biological behavior of NeuroEndocrine Neoplasias (NENs). Only a few genes in hereditary cases of NENs have been identified.

Although a few novel tumor suppressor genes have been identified and the intergenic cooperation models need to be integrated with these new findings, it should be considered that some of the genes involved in NENs tumorigenesis have complex in functions in genomic integrity.

Furthermore, for some NENs, other mechanisms should be considered, e.g., splicing deregulation, while for others, scenarios of a permissive chromatin landscape, the dysregulation of endogenous retrovirus sequences or the modification of cellular plasticity, due to the alteration of the expression of specific miRNAs, have been described.

The complexity of NENs tumorigenesis emphasizes how focusing on the identification of a small number of genetic events does not allow us to profile all of the cases and explore all of the therapeutic potential inherent in the other mechanisms.

In summary, the studies here offer a glance at the baffling puzzle of tumorigenesis and suggest how the great improvements allowed by NGS technologies, in terms of knowledge and clinical management, have opened up new possibilities and raised new questions. Hence, the need to integrate them with other methodological approaches is a difficult task due to the mapping of the dynamic complexity of cancer evolution.
